# Building the plane while it’s flying: implementation lessons from integrating a co-located exercise clinic into oncology care

**DOI:** 10.1186/s12913-022-08607-w

**Published:** 2022-10-06

**Authors:** Mary A. Kennedy, Sara Bayes, Robert U. Newton, Yvonne Zissiadis, Nigel A. Spry, Dennis R. Taaffe, Nicolas H. Hart, Daniel A. Galvão

**Affiliations:** 1 Exercise Medicine Research Institute, Edith Cowan University, 270 Joondalup Drive, Joondalup, Perth, WA 6027 Australia; 2 School of Medical and Health Sciences, Edith Cowan University, 270 Joondalup Drive, Joondalup, Perth, WA 6027 Australia; 3 School of Nursing, Midwifery and Paramedicine, Australian Catholic University, Fitzroy, VIC Australia; 4 School of Nursing and Midwifery, Edith Cowan University, Perth, WA Australia; 5 GenesisCare, Perth, WA Australia; 6 Caring Futures Institute, College of Nursing and Health Sciences, Flinders University, Adelaide, SA Australia

**Keywords:** Cancer, Physical activity, Organizational change, Barriers, Chemotherapy, Radiotherapy

## Abstract

**Background:**

Despite its therapeutic role during cancer treatment, exercise is not routinely integrated into care and implementation efforts are largely absent from the literature. The aim of this study was to evaluate a strategy to integrate the workflow of a co-located exercise clinic into routine care within a private oncology setting in two clinics in the metropolitan region of Western Australia.

**Methods:**

This prospective evaluation utilised a mixed methods approach to summarise lessons learned during the implementation of an integrated exercise workflow and supporting implementation plan. Data collection was informed by the RE-AIM (Reach, Effectiveness, Adoption, Implementation, Maintenance) framework. Reports detailing utilisation of the exercise service and its referral pathways, as well as patient surveys and meeting minutes documenting the implementation process informed the evaluation.

**Results:**

The co-located exercise service achieved integration into routine care within the clinical oncology setting. Patient utilisation was near capacity (reach) and 100% of clinicians referred to the service during the 13-month evaluation period (adoption). Moreover, ongoing adaptations were made to improve the program (implementation) and workflows were integrated into standard operating practices at the clinic (maintenance). The workflow performed as intended for ~70% of exercise participants (effectiveness); however, gaps were identified in utilisation of the workflow by both patients and clinicians.

**Conclusion:**

Integration of exercise into standard oncology care is possible, but it requires the ongoing commitment of multiple stakeholders across an organisation. The integrated workflow and supporting implementation plan greatly improved utilisation of the co-located exercise service, demonstrating the importance of targeted implementation planning. However, challenges regarding workflow fidelity within and across sites limited its success highlighting the complexities inherent in integrating exercise into clinical oncology care in a real-world setting.

**Supplementary Information:**

The online version contains supplementary material available at 10.1186/s12913-022-08607-w.

## Background

The role of exercise for people living with cancer has rapidly evolved over the last 30 years. Prior to the first known randomised controlled research trial in the late 1980s demonstrating significant promise in cancer patients, exercise was considered something to avoid during cancer treatment [1] with ‘rest therapy’ being oncologists’ standard recommendation for their patients [2]. Interest in the therapeutic potential of exercise grew steadily over the next 20 years. The evidence base established during this time led to the field’s next milestone in 2009 and 2010 when the first exercise guidelines for cancer survivors were published [3, 4]. This guidance allowed practitioners to provide recommendations to cancer patients regarding what exercise was safe and effective, establishing that ‘some activity is better than none’. In the decade since, the evidence demonstrating the role of exercise in cancer has grown exponentially, prompting an update to the guidelines in 2019 [5, 6], which established the therapeutic role of exercise in cancer, and made clear the need to ensure the evidence was being used in clinical practice. This new understanding of the progress made in the field prompted a call to action for the clinical oncology community to engage in efforts to ensure exercise is provided as routine care for people with cancer [7] and has prompted a multidisciplinary team of international experts to propose an agenda to make exercise standard practice in oncology [8].

Establishing an evidence base for exercise in oncology is just the first step toward translating research into practice. Integrating new practices into healthcare is notoriously difficult and time consuming [9]. The emerging field of implementation science has evolved to provide systematic, evidence-informed approaches to more efficiently change practices within healthcare. A ‘one-size-fits-all’ approach is not feasible [10] as a core tenant of implementation science is the need to design strategies to meet the specific needs of the context where they will be implemented [11]. A scoping review of implementation barriers to integrating exercise into oncology care highlights the multitude of obstacles that exist across a health system [12]. The review identified nearly 250 unique barriers that had been reported by oncology clinicians, patients, and healthcare systems internationally, which provided a generalized map of potential issues for implementation for the field broadly. However, given the wide variation in models of oncology care delivery (e.g., private vs. public, rural vs. urban, chemotherapy vs. radiotherapy) healthcare systems working toward exercise integration will need to identify the barriers relevant in their own context in order to create a viable implementation plan.

**Fig. 1:**
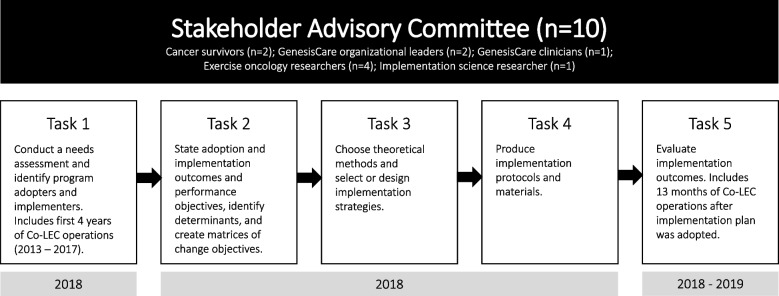
Outline of Implementation Mapping process

Recent examples in exercise oncology demonstrate the process of contextualizing implementation barriers [13–15], including the GenesisCare co-located exercise clinic (Co-LEC) in Western Australia [16]. The Co-LEC is an exercise clinic embedded within a private oncology treatment centre. The Implementation Mapping (IM) process [17] (Fig. 1) was used to develop an implementation plan to guide implementation of the Co-LEC into standard practice at GenesisCare. Tasks 1 – 4 have been previously described [16, 18, 19]. Briefly, Task 1 of the process consisted of an evaluation of existing practices and highlighted utilisation challenges among patients and staff due to implementation gaps [16, 18]. Specifically, the referral pathway was not clear, the workflow was not integrated, and there was no sustainable financial plan in place to support the program for the long term. These issues resulted in the Co-LEC being underutilised by patients and oncologists. This information was used in Tasks 2 – 4 to inform the development of an integrated workflow supported by a contextually specific implementation plan to integrate the service into routine care at GenesisCare (described in methods) [19]. This plan was co-developed with multiple stakeholder groups, incorporated several implementation strategies, and defined specifically who needed to do what to operationalize the process.

This evaluation study represents Task 5 of the IM process (Fig. 1). The aim was to evaluate the implementation outcomes of the Co-LEC after operationalization of the implementation plan. As continued effort is made toward understanding how to embed exercise into standard practice in oncology, it is critical to share experiences of what does and does not work regarding implementation across various contexts. Though not directly transferable, the experiences of GenesisCare’s integration of the Co-LEC into routine care will help create a blueprint of best practices to guide the broad initiative to make exercise standard practice in oncology [8].

## Methods

### Study design

This mixed-methods evaluation study was conducted between December 1, 2018 and December 31, 2019. All clinical data extracted from GenesisCare records were de-identified before being shared with the study team. Ethics approval was provided by Edith Cowan University’s Human Research Ethics Committee (ID: 20888 KENNEDY) to ensure it was planned and carried out according to relevant guidelines and regulations. All participants provided informed consent to participate in the study. The RE-AIM (Reach, Effectiveness, Adoption, Implementation, Maintenance) framework was chosen to guide this evaluation given its focus on research translation and its ability to provide a direct comparison to results from the initial evaluation of the Co-LEC, prior to the development of a supporting implementation plan [12]. RE-AIM evaluates a program using five constructs to provide a comprehensive perspective of a program’s success (Table 1) [20].

**Table 1:** Components of RE-AIM evaluation framework

Construct and definition applied for this study	Questions addressed	Data sources used
**Reach** The number and proportion of people who participated in the Co-LEC	1. How many people participated in an initial assessment at the Co-LEC compared to how many people received treatment at GenesisCare? 2. How many people participated in an in an initial assessment at the Co-LEC compared to the capacity of the service? 3. Why did people decline participation in the Co-LEC?	Co-LEC records Routinely collected GenesisCare data
**Effectiveness** The performance of the Co-LEC workflow in practice	4. Did the workflow perform as intended? If not, why?	Billing records Co-LEC records Exercise working group notes Patient satisfaction surveys
**Adoption** The number and proportion of key staff who participated in the Co-LEC workflow	5. How many oncologists participated in referrals to the exercise clinic? 6. What proportion of participants overall were referred by each practitioner? 7. Did the supporting staff execute the workflow as expected? If not, why?	Co-LEC records Exercise working group notes
**Implementation** Adaptations made to Co-LEC workflow or its supporting functions	8. What adaptations were made to the Co-LEC workflow or its supporting functions?	Co-LEC records Exercise working group notes
**Maintenance** The extent to which the program became part of routine organisational practices	9. Did the Co-LEC become institutionalised as part of routine organisational practices?	Exercise working group notes

*Abbreviations*: *Co-LEC* Co-located exercise clinic, *RE-AIM* Reach, Effectiveness, Adoption, Implementation, Maintenance

### Setting

This study was conducted at two GenesisCare outpatient oncology clinics (i.e., Clinic 1 established 2013 and Clinic 2 established 2017) approximately 28 km apart in the metropolitan region of Perth, Western Australia. The clinics are part of GenesisCare’s global network of private health care clinics and were the only locations with a Co-LEC at the time the study commenced. Though the clinics were managed by separate centre leaders responsible for overseeing day-to-day operations of their respective clinic (including the Co-LEC), both were part of a network of regional clinics managed by the same leadership team (general and operations managers). Nearly 40% (5/13) of oncologists worked across the two locations and the clinic management teams regularly collaborated on projects, including the implementation of the Co-LEC.

### Program description

#### Co-LEC integrated workflow

An integrated workflow was developed to shift the Co-LEC to become an ‘opt-out’ service for patients whereby exercise was considered a routine part of cancer treatment. All workflow processes were designed using existing systems at GenesisCare and management informed relevant staff that operations related to the Co-LEC workflow were to be considered a part of their job responsibilities.

**Fig. 2:**
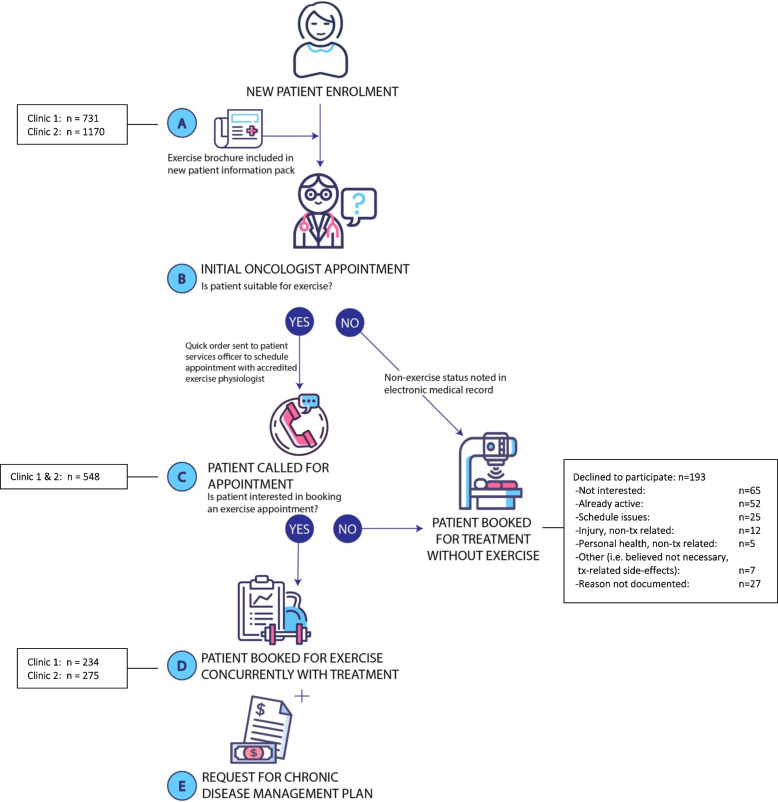
Integrated workflow for the co-located exercise clinics

The integrated workflow addressed each phase of the patient journey (Fig. 2). Before attending their initial appointment at GenesisCare, patients received a brochure describing the Co-LEC service and the overall benefits of exercise during treatment (Fig. 2, Step A). During the initial appointment, where the decision regarding whether to commence treatment was made, the oncologist discussed exercise and the Co-LEC service with the patient. When the decision to commence treatment was noted in the electronic medical record (EMR), the oncologist was prompted to tick a box to declare whether the patient was fit for exercise (Fig. 2, Step B). Ticking the ‘suitable for exercise’ box triggered a ‘quick order’ for an administrative staff member (i.e., patient services officer (PSO)) to call the patient to book an initial assessment with an Accredited Exercise Physiologist (AEP) at the Co-LEC (Fig. 2, Step C). If the patient declined the appointment, they continued with treatment as usual. If the patient was interested in exercise, the PSO booked an initial assessment with an AEP at the Co-LEC timetabled with the patient’s treatment time (Fig. 2, Step D). Scheduling practices for the Co-LEC were integrated into GenesisCare’s electronic system so that exercise appointments were visible along with all treatment-related appointments in a patient’s chart.

An AEP with experience creating exercise programs for people with cancer was employed through GenesisCare to see patients at the Co-LEC. The position was 0.6 full-time equivalent (FTE) and was split between the two clinics in accordance with patient treatment numbers. The AEP spent one full day per week at clinic 1 (8 h) and two full days per week at clinic 2 (16 h). Initial exercise assessments were conducted by the AEP. There were 4 assessment appointments available per week at clinic 1, setting the total capacity over the 13-month (56 weeks) evaluation period at 224 new patients. Clinic 2 offered 8 appointments per week, setting the capacity at 448 over 13 months. The service was offered at no additional cost to patients; however, a Medicare billing option was introduced for patients who could obtain a chronic disease management plan (CDMP) [21] from their general practitioner (GP). This billing structure was designed to cover the cost of fully integrating the service without having to charge patients an additional out-of-pocket expense. Every Co-LEC appointment with the AEP was billable if a patient obtained a CDMP. At the time of the evaluation Medicare provided $53.80 (AUD) per appointment for a maximum of 5 visits per year (the number of visits determined by their GP) [21]. The CDMP payment procedure was described in the Co-LEC brochure that patients received prior to their initial oncologist appointment and all staff were educated about the process during their respective orientation to the new workflow. Additionally, the AEP requested a CDMP from the patient at their initial assessment appointment (Fig. 2, Step E). The GenesisCare billing department introduced a protocol into their weekly workflow to track and bill for CDMPs received by Co-LEC patients.

### Implementation plan

The integrated workflow was supported by a multifaceted implementation plan developed by Co-LEC key stakeholders using the implementation mapping process to ensure it was contextually appropriate [13]. Eight discrete strategies from the expert recommendations for implementing change (ERIC) [22] were operationalised (Table 2). An exercise implementation working group (exercise working group) was established to oversee the project. Group members included the operations manager and centre leaders for clinics 1 and 2. An implementation advisor was employed to advise the project for the first six months (December 2018 to May 2019) and participated as part of the working group during that time period.

**Table 2:** Operationalisation of implementation strategy

ERIC category Implementation strategy	Operationalisation of strategy
**Use evaluative and iterative strategies**	
Audit and provide feedback	Key outcome measures were identified (total # of new patient appointments, % utilisation, patients queued for assessment, $ earned) and tracked weekly, A team of key stakeholders for the exercise clinic was identified, which included the operations manager, centre leaders, exercise physiologist, implementation advisor, and marketing manager. The team scheduled weekly updates to review the data and address any critical issues that arose.
**Develop stakeholder interrelationships**	
Identify and prepare champions	A senior oncologist who had expressed a strong interest in the Co-LEC during the evaluation process and worked across both sites was asked to join a strategic exercise working group to provide clinical insight into the operational decisions of the clinic. She also served as a liaison between the business and clinical staff to discuss
Use an implementation advisor	An implementation advisor was included as part of the key stakeholder and strategic working group teams for the first 6 months of the project.
**Train and educate stakeholders**	
Conduct educational meetings	Oncologists: A meeting was arranged prior to the launch of the Co-LEC to provide a detailed overview of the workflow and roles for all oncologists. Administrative staff: Each centre organised an orientation to the Co-LEC for relevant administrative staff. Ad-hoc sessions were scheduled with the administrative staff as new procedures were introduced.
Develop educational materials	Information sheets that specified workflow and procedures for all administrative staff in relation to the Co-LEC were created and shared with staff as appropriate. These were updated as needed.
**Utilise financial strategies**	
Access new funding/use other payment	Medicare CDMPs were utilised to help cover the costs of running the Co-LEC. The billing team created a workflow to track and bill for Medicare-reimbursable sessions on a weekly basis.
**Change infrastructure**	
Change record systems	The EMR was updated to allow exercise appointments to be scheduled and tracked as a part of a patient’s daily treatment schedule. CDMPs were uploaded and attached to patient’s records.
**Support clinicians**	
Revise professional roles	The AEP was employed through GenesisCare; the Co-LEC responsibilities were written into the job descriptions for all relevant administrative roles, including centre leaders, PSOs and billing staff.

*Abbreviations*: *AEP* Accredited Exercise Physiologist, *CDMP* Chronic disease management plan, *Co-LEC* Co-located exercise clinic, *EMR* Electronic medical record, *ERIC* Expert recommendations for implementing change, *PSO* Patient services officer, *#* Number, *$* Dollar, *%* Percent

### Data sources

#### GenesisCare clinic records

The data specialist at GenesisCare extracted the total number of new patients who received treatment during the evaluation period at the two clinics.

### Co-LEC reports

#### Booking calls

A report detailing the PSO calls to book Co-LEC appointments was created within GenesisCare’s EMR. The report included the patient’s name, the referring oncologist, and whether the patient accepted or declined the appointment. If a patient declined the appointment, PSOs were instructed to take notes to explain why. This report captured only responses from patients who came to the Co-LEC via the integrated workflow (Fig. 2). It did not capture patients who came via different pathways (e.g., self-referred) when the workflow did not perform as intended.

#### Appointments

The Co-LEC clinic schedule within GenesisCare’s EMR system was searched to identify all patients scheduled for an initial assessment during the evaluation period. A report of appointments scheduled, whether they were attended or cancelled, and relevant scheduling notes was generated by a GenesisCare staff member.

#### Billing records

All CDMPs associated with the Co-LEC were recorded by the billing team. A report detailing CDMPs on file and their billing status was created.

### Patient surveys

All GenesisCare patients were offered the opportunity to complete a patient satisfaction survey (Supplementary File 1) at the end of their treatment. A report detailing survey comments relevant to the exercise service was created.

### Exercise working group meeting minutes

An exercise working group was established in October 2018 to begin planning for the integration of the service. Initial meetings were called with the oncologists and PSOs to inform them about their roles in the integrated workflow and clarify the expectation that the Co-LEC would be accommodated as part of their usual job responsibilities. PSO meetings with the exercise working group were planned monthly (*n* = 13) and oncologist meetings were planned annually (*n* = 2) between December 2018 and December 2019. Meeting minutes were recorded and formed a data set.

### Data analysis

Quantitative data were analysed using Jamovi (version 1.2 Sydney, Australia) [23] and are reported according to the questions identified in Table 1. Simple generic thematic analysis was used to code and categorise the open notes responses in booking records [24]. Qualitative data from patient surveys and exercise working group meeting minutes were analysed using a descriptive approach designed for practitioners and policy makers whereby standard language is used to describe facts without the need for abstract theorising [25]. A deductive approach to analysis was used, focused on identifying common challenges related to each RE-AIM construct. Descriptive summaries were created through interpretation of survey and exercise working group data. Data analysis was primarily undertaken by MAK with review and input from the other authors. The combined audit summaries were developed by MAK, with input from SB. Results are reported descriptively, in-line with each construct of the RE-AIM framework.

## Results

### Construct 1: reach

#### Demographics

Demographic features of the Co-LEC participants are provided in Table 3. Participants ranged in age from 30 to 92 years and over half (56%) were female. Overall, people being treated for nineteen different types of cancer participated in the Co-LECs, though breast and prostate cancers accounted for the majority [37% (*n* = 189) and 20% (*n* = 103) respectively]. Demographic information was not available for the people who did not use the Co-LEC.

**Table 3:** Characteristics of patients who participated in the co-located exercise clinics

	No. (%)	
	Clinic 1 (***n*** = 234)	Clinic 2 (***n*** = 275)
**Age, median [IQR], years**	**66.0 [55.0–73.0]**	**58.0 [57.0–74.5]**
< 39	7 (3.0)	10 (3.6)
40-49	25 (10.7)	26 (9.5)
50-59	47 (20.1)	43 (15.6)
60-69	62 (26.5)	69 (25.1)
70-79	71 (30.3)	100 (36.4)
80+	22 (9.4)	27 (9.8)
**Sex**		
Male	86 (36.8)	137 (49.8)
Female	148 (63.2)	138 (50.2)
**Cancer type**		
Breast	109 (46.6)	80 (29.1)
Prostate	42 (17.9)	61 (22.2)
Lung	12 (5.1)	7 (2.5)
Colorectal	6 (2.6)	19 (6.9)
Endometrial	7 (3.0)	10 (3.6)
Head and neck	15 (6.4)	23 (8.4)
Melanoma	12 (5.1)	12 (4.4)
Metastatic	8 (3.4)	13 (4.7)
Other^a^	23 (9.8)	50 (18.1)

^a^Cases that are fewer than 5 (i.e., 1-4) are listed in other category for privacy, which includes anal, appendix, bile duct, bladder, brain, cervical, non-Hodgkin lymphoma, oesophageal, ovarian, pancreatic, stomach

### Clinic utilisation

#### Clinic 1

**Fig. 3:**
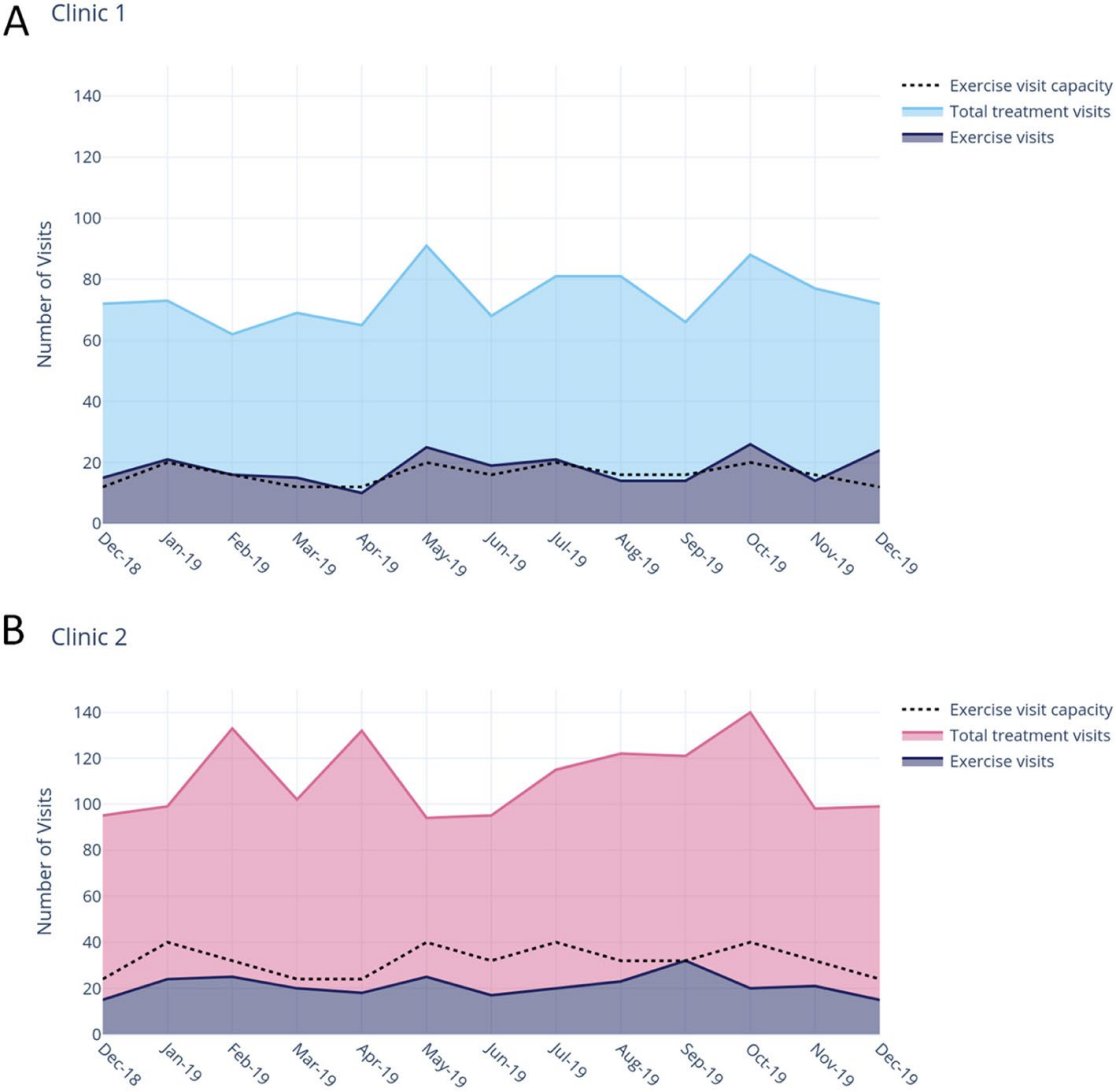
Reach and capacity of co-located exercise clinics

Over the 13-month evaluation period, 731 patients commenced a course of treatment (radiotherapy, chemotherapy or combination) at GenesisCare’s clinic 1 location. Of these, 234 attended an initial assessment at the Co-LEC. The clinic operated at 104% of initial capacity, reaching 32% of all patients receiving treatment (Fig. 3A).

#### Clinic 2

One thousand one hundred seventy (*n* = 1170) patients commenced radiotherapy treatment at clinic 2 during the evaluation period with 275 attending an initial assessment at the Co-LEC. This exercise clinic operated at 61% of initial capacity, reaching 24% of all patients receiving treatment (Fig. 3B).

#### Non-utilisation

The PSOs made 548 calls to book patients into the Co-LEC (Fig. 2, Step C); 35% (*n* = 193) of patients declined to participate. Open notes were available for 86% (*n* = 166) of the patients who declined an appointment. The leading responses were represented in three categories: generic ‘not interested’ (*n* = 65, 34%), ‘already active or enrolled in a similar program’ (*n* = 52, 27%), and ‘schedule or logistical issues’ (*n* = 25, 13%). Health-related concerns were noted in 11% (*n* = 21) of cases; however, very few (*n* = 4) were due to treatment-related side-effects. Most (*n* = 12) were a result of a previous injury or illness.

### Construct 2: effectiveness

#### Scheduling the initial appointment

Forty percent (*n* = 295) of patients who received treatment at clinic 1 were offered exercise via a PSO call (Fig. 2, Step C). At clinic 2, 22% (*n* = 253) of patients were called. Any additional attempts to inform patients about the Co-LEC were not captured.

Of the 234 patients who attended the Co-LEC at clinic 1, 183 (78%) were booked by a PSO (Fig. 2, Step D). At clinic 2, PSOs booked 59% (*n* = 161) of the 275 exercise appointments. All other appointments were booked outside of the workflow.

#### Obtaining a CDMP

A CDMP was provided by 27% (*n* = 136) of the 509 patients who attended an initial assessment during the evaluation period (Fig. 2, Step E). The billing records did not specify which Co-LEC the patient attended.

#### Capacity

Data from two sources (exercise working group meeting notes and patient surveys) clearly indicated that the Co-LEC’s limited capacity was a concern for patients, oncologists, and PSOs. Patients reported the “limited availability” of the exercise physiology appointments. A group of 6 oncologists reported a need for “more availability” for their patients, and PSOs reported the lack of available appointments as a major challenge for the service, noting a lack of clarity about what to do when the exercise schedule was overbooked.

### Construct 3: adoption

#### Oncologist engagement

All oncologists agreed to participate in the integrated workflow when it was introduced by the exercise working group. Booking records confirmed 100% engagement from the 13 oncologists. The clinic 1 Co-LEC received 295 referrals, with most (72%, *n* = 211) from two oncologists. Clinic 2’s Co-LEC received 253 referrals. Two oncologists were responsible for 46% (*n* = 116) of the referrals and 4 others each contributed approximately 10% to the total.

#### PSO engagement

PSOs found it difficult to accommodate the additional work that Co-LEC scheduling added to their usual workload. They noted it took “a lot of time”, especially when trying to timetable exercise and treatment appointments. One PSO explained that “exercise is the first thing to go” when, for example, the treatment schedules were overbooked. These staff struggled to keep up with their usual daily responsibilities during busy periods and could not manage the additional Co-LEC-related workload at these times.

### Construct 4: implementation

**Fig. 4:**
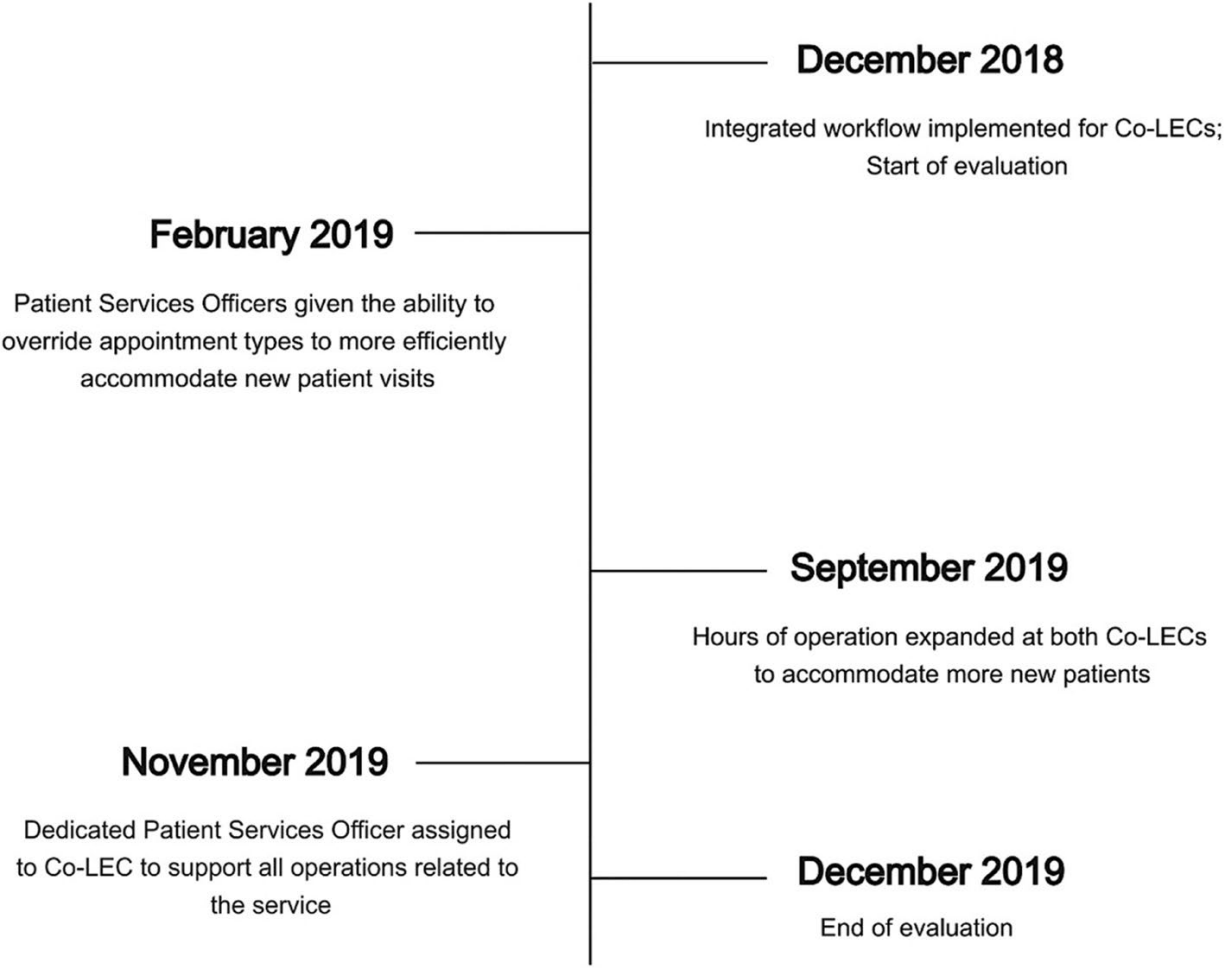
Timeline of implementation adaptations made at the co-located exercise clinics

Adaptations in two areas were made to overcome issues that were seen to be causing the workflow to run inefficiently: scheduling and staffing (Fig. 4).

#### Scheduling

Lack of capacity for initial assessments was raised as a challenge by all workflow stakeholders (i.e., patients, oncologists, PSOs) from the beginning of integration. An adjustment to the scheduling process was made in February 2019 to allow PSOs to override appointment slots intended for ongoing exercise appointments and allocate them to new patients with approval from the AEP. This allowed the AEPs time to be utilised most effectively relative to new patient versus ongoing patient demand and resulted in Clinic 1 operating above initial capacity.

#### Staffing

From September 2019, additional AEPs were hired to expand the hours of operation across both clinics as an additional way to combat the challenges related to capacity. The added hours allowed for 2 more assessment visits per week at each clinic.

Concerns about the extra work created for PSOs by the Co-LEC scheduling process were continually raised to the exercise working group. In response, from November 2019 a Co-LEC PSO role was created to support all operations related to the exercise service. This person worked across both Co-LECs to complete all bookings, billing, and administrative needs related to the service. Additionally, the responsibility for informing patients about the CDMP shifted to the person in this role, whose remit was to request a CDMP during the booking phone call.

### Construct 5: maintenance

The program was maintained for seven months after the implementation advisor stopped working with the clinics. All elements of the integrated workflow continued to operate. The exercise working group continued to meet regularly and had expanded to include the state’s quality and safety manager and marketing director. Additionally, the exercise working group had undertaken a project to formally document the standard operating procedures of the Co-LEC. In December 2019, the focus areas for the exercise working group were expansion of the Co-LEC’s billing options, continued integration of Co-LEC operations, and identification of future research opportunities relevant to the Co-LEC’s implementation efforts.

## Discussion

This study used the RE-AIM framework to evaluate the integration of a co-located exercise clinic into the standard operations of a private cancer treatment clinic. The evaluation encompassed the first 13 months of the Co-LEC’s operations across two separate clinics, with implementation support provided for the first six months. Three important findings were revealed from this evaluation. First, implementation planning is important to showcase the true potential of an intervention. Second, while exercise has a therapeutic role in medicine, it does not fit into the traditional medical model from a systems point of view. This systems mismatch is an important barrier to integration. Third, establishing best practices for integration of exercise into oncology care is a dynamic process that requires resources, time, and ongoing attention. Significant buy-in from healthcare organisations is critical to its success.

Targeted implementation planning can help programs overcome logistical barriers masking their potential impact. Over its first ~4 years of operation, the clinic 1 Co-LEC achieved a reach of 12% [16]. This outcome aligns with similar efforts in Australia by Dennett et al. [14, 15]. Their efforts to embed exercise into a co-located cancer unit achieved a reach of 10% [14] and their telerehabilitation service reached just 9% of patients [15]. The introduction of the integrated workflow and supporting implementation strategies increased the reach of the clinic to 32% over the 13-month evaluation period. Moreover, this nearly three-fold increase was limited by the program’s capacity. The decision to start with a limited capacity program at each Co-LEC location reflects the challenges of working with real-world clinics with business income goals that must be realised [26]. GenesisCare had an organisational responsibility to demonstrate buy-in from oncologists and patients and re-assess before expansion. Oncologist buy-in was evident from effective use of the oncologist-initiated workflow. In our previous evaluation [16] oncologist referrals only accounted for 21% of Co-LEC new patient visits. In contrast, this evaluation found 78% of patients at clinic 1 and 59% at clinic 2 were referred to the Co-LEC via the oncologist-initiated workflow. Moreover, concerns about a lack of clarity around the referral process expressed in the initial evaluation were not evident, instead oncologists were most concerned about increasing the capacity of the service to accommodate their patients more effectively. One unexpected finding was the high proportion (35%) of patients who declined to participate in the Co-LEC. This proportion of decline is much higher than the range of 8 – 11% in similar Australian models reported by Dennett et al. [13, 15], and warrants further investigation. The comparison of the service before and after implementation planning demonstrates the ability of contextually specific plans to improve a program’s success and reinforces the need to incorporate implementation planning into intervention planning [27]. This is especially important for exercise oncology programs today as the field is at a critical stage of establishing its place in standard oncology care. Assessing programs that have not planned for effective implementation runs the risk of diluting the potential value they may offer.

Professionals in the field of exercise oncology have made great strides in establishing exercise as an effective therapy to address multiple health-related side effects of cancer [5, 6], but the operational components required to provide exercise services for people with cancer are not yet part of traditional medical systems. This incongruence is an underappreciated barrier impeding the integration of exercise into standard oncology care. A primary example of the mismatch in the provision of exercise services versus the provision of traditional medical services is the financial operation of each. Finances have been noted as a critical concern for the sustainability of exercise oncology programs [16, 28] in large part because exercise physiology services are not billable using traditional healthcare mechanisms [29]. Most countries do not have an option for healthcare organisations to bill visits with exercise physiologists [29], and for those that do (such as Australia) the procedures are separate to standard Medicare billing practices [30]. To offer the CDMP option to patients, GenesisCare’s billing department had to create a new protocol to ensure exercise visits could be billed. This protocol was linked to the scheduling system, which also had to be created within GenesisCare systems. Moreover, the process for a patient to obtain a CDMP required a separate visit to their GP. Despite the fact the CDMP was described in the patient brochure (Fig. 2, Step A) and all staff were told about the payment option for patients, the AEP appeared to be the only person who took the time to fully discuss the CDMP option with patients (Fig. 2, Step E). However, because that discussion occurred during the initial assessment, the assessment appointment was not billable as CDMPs cannot be billed retrospectively [21]. This likely accounts for the relatively low (27%) uptake of CDMPs for initial assessments reported in this evaluation. Moreover, this low uptake level suggests that an exercise service cannot be solely reliant on CDMPs for financial viability. Still, It is widely accepted that change within healthcare is extremely difficult [8]. For an organisation to design, implement, and adopt new practices within 13 months suggests GenesisCare has the attributes of a learning organisation [31] that will be required to integrate exercise into routine clinical care in oncology.

Organisational support is vital to the success of exercise in standard oncology care. The integrated workflow and implementation plan described in this evaluation required buy-in and support of the GenesisCare organisation for both its adoption and maintenance. As described earlier, the lack of established systems for exercise in medicine required a substantial amount of work to create pathways for its adoption. Every component of the workflow required dedicated time and resourcing to make operational. Additionally, vast institutional knowledge was necessary to ensure new systems were compatible with established practices within the clinics. Once created, successful execution of the workflow depended on the participation of multiple stakeholder groups across this dynamic organisation. The workflow appeared to be a good organisational fit as evidenced by the 100% oncologist adoption rate; however, it had issues regarding its effectiveness. The booking records suggest approximately 30% of patients who attended the Co-LEC were not booked via the pathway outlined in the workflow. While these alternative routes were not captured by the reports, the fact they exist exposes issues that need to be addressed as the program continues to make adaptations. Moreover, the difference in effective execution of the workflow between clinic 1 and clinic 2 (78% vs. 59% respectively) suggests a difference in staff engagement between clinics. This difference could also reflect the leadership at each location, as leadership attitude and style has an important influence in the adoption of new programs [32]. Understanding the complexity regarding adoption of a new system is important as the field of exercise oncology works to create resources for universal dissemination. For example, the *Moving Through Cancer* searchable registry aims to provide an exercise referral resource for healthcare providers to assist in achieving the goal of making exercise a routine part of cancer care [7, 33]. While this resource fills a critical gap in the field, it must be adopted and integrated into routine practice to be effective. Our evaluation suggests effective utilisation of new resources, such as the registry, requires context-specific, systematic integration at an organisational level.

Beyond adoption, organisational support is also critical for program maintenance. The IM process used to develop the Co-LEC implementation strategy is an iterative process [17], with most programs requiring multiple adaptations before achieving a good fit within an individual context [10]. Program adaptation is a resource-intensive process that takes time, money, and the openness of staff to make changes. An organisation’s capacity and willingness to accommodate a program’s evolving needs is critical for its successful maintenance (i.e. implementation success) [34]. For example, it took one year to identify the need for and create a PSO role to more effectively support the Co-LEC. This demonstrates GenesisCare’s commitment to the maintenance of their exercise program. The development of an implementation plan should be viewed as the beginning, not the end, of a program’s implementation efforts. The plan is a living document that requires ongoing investment. Understanding this is especially important in exercise oncology, where systems are being built for programs at the same time they are being tested.

### Strengths and limitations

A major strength of this evaluation was its real-world setting, which exposed practical issues translatable to others trying to employ exercise into a clinical setting. Additionally, the evaluation was guided by the RE-AIM evaluation framework, used a mixed-methods approach to provide context to the quantitative data and incorporated implementation strategies from the ERIC project. However, the results are limited to the private clinical setting. Important differences may be present in public settings, such as the socioeconomic status of people receiving care and timeliness of service [35]. Finally, because the workflow was entirely new, the reporting structures also needed to be established and were not complete by the time of this evaluation. As a result, some details could not be reported that would have added to the results, such as how many patients were marked ‘not suitable’ for exercise or how alternative pathways to the integrated workflow emerged.

## Conclusion

Integration of exercise into standard oncology care is possible but requires the ongoing efforts of multiple stakeholders across an organisation. The integrated workflow and supporting implementation plan greatly improved utilisation of the Co-LEC across two clinic locations demonstrating the importance of targeted implementation planning. However, challenges regarding workflow fidelity within and across sites limited the success of the service. This evaluation highlights the complexities inherent in integrating exercise into clinical care in a real-world setting.

## Supplementary Information

**Additional file 1.** Exercise service patient satisfaction survey.

## Data Availability

Original data are not available due to agreements between GenesisCare and Edith Cowan University but are available from the corresponding author on reasonable request.
