# Supplementary material for: Building the plane while it’s flying: implementation lessons from integrating a co-located exercise clinic into oncology care
Source: BMC Health Serv Res. 2022 Oct 6;22:1235. doi: 10.1186/s12913-022-08607-w (PMC9535901; doi:10.1186/s12913-022-08607-w)
Supplement: Supplementary file 1 — Additional file 1. Exercise service patient satisfaction survey. [file 12913_2022_8607_MOESM1_ESM.docx]

Supplementary File 1. Exercise service patient satisfaction survey

1. How did you hear about the GenesisCare exercise service?
2. When you heard GenesisCare had an exercise program, what did you expect it would be like?
3. What were the things you liked about the service?
4. What areas do you feel could be improved about the service?
5. Are you still exercising at GenesisCare?
6. Anything else you would like to share about your experience with the exercise service?
